# Clinical Outcomes for Patients With Anosmia 1 Year After COVID-19 Diagnosis

**DOI:** 10.1001/jamanetworkopen.2021.15352

**Published:** 2021-06-24

**Authors:** Marion Renaud, Claire Thibault, Floriane Le Normand, Emily G. Mcdonald, Benoît Gallix, Christian Debry, Aina Venkatasamy

**Affiliations:** 1 Otorhinolaryngology Department, University Hospitals of Strasbourg, Strasbourg, France; 2 Clinical Practice Assessment Unit, Department of Medicine, McGill University, Montreal, Quebec, Canada; 3 Institut Hospitalo-Universitaire-Strasbourg, Strasbourg, France; 4 Department of Radiology, McGill University, Montreal, Quebec, Canada; 5 Faculte de Medecine, Universite de Strasbourg, Strasbourg, France; 6 Streinth Laboratory (Stress Response and Innovative Therapies), Inserm UMR_S 1113, Interface Recherche Fondamental et Appliquee à la Cancerologie, Strasbourg, France

## Abstract

This cohort study examines the clinical course and prognosis of patients with COVID-19–related anosmia for 1 year after diagnosis.

## Introduction

Since the pandemic was declared in early 2020, COVID-19–related anosmia quickly emerged as a telltale sign of infection.^1,2^ However, the time course and reversibility of COVID-19–related olfactory disorders, which may persist and negatively affect patients’ lives, require further study. To clarify the clinical course and prognosis, we followed a cohort of patients with COVID-19–related anosmia for 1 year and performed repeated olfactory function evaluations for a subset of patients.

## Methods

 This cohort study follows the Strengthening the Reporting of Observational Studies in Epidemiology (STROBE) reporting guideline. Participants provided written informed consent. The study was approved by the ethics committee of the University Hospitals of Strasbourg.

In April 2020, we published a study^1^ about a cohort of patients with polymerase chain reaction–proven COVID-19 with acute smell loss (lasting >7 days). Over the course of 1 year, at 4-month intervals, patients were asked to complete a survey, and their olfactory function was assessed by psychophysical testing (the threshold and identification tests; Sniffin’ Sticks Test; Burghardt).^3^ Hyposmic or anosmic patients were followed until objective olfactory recovery (normal results were defined as those at or above the 10th percentile). Data analysis was performed from June 2020 to March 2021.

## Results

We evaluated 97 patients (67 women [69.1%]; mean [SD] age, 38.8 [11.5] years) with acute smell loss beyond 7 days. Of these patients, 51 (52.6%) underwent both subjective and objective olfactory test, and 46 (47.4%) underwent subjective assessment alone (Figure). After subjective assessment at 4 months, 23 of 51 patients (45.1%) reported full recovery of olfaction, 27 of 51 patients (52.9%) reported partial recovery, and 1 of 51 patients (2.0%) reported no recovery. On psychophysical testing, 43 of 51 patients (84.3%) were objectively normosmic, including 19 of 27 (70.0%) who self-evaluated as only partially recovered (all patients who self-reported normal return of smell were corroborated with objective testing) (Table). The remaining 8 patients (15.7%) with persistent subjective or objective loss of smell were followed up at 8 months, and an additional 6 patients became normosmic on objective testing. At 8 months, objective olfactory assessment confirmed full recovery in 49 of 51 patients (96.1%). Two patients remained hyposmic at 1 year, with persistent abnormalities (1 with abnormal olfactory threshold and 1 with parosmia causing abnormal identification). Among those who underwent subjective assessment alone, 13 of 46 patients (28.2%) reported satisfactory recovery at 4 months (7 with total and 6 with partial recovery), and the remaining 33 patients (71.7%) did so by 12 months (32 with total and 14 with partial recovery).

**Figure.**
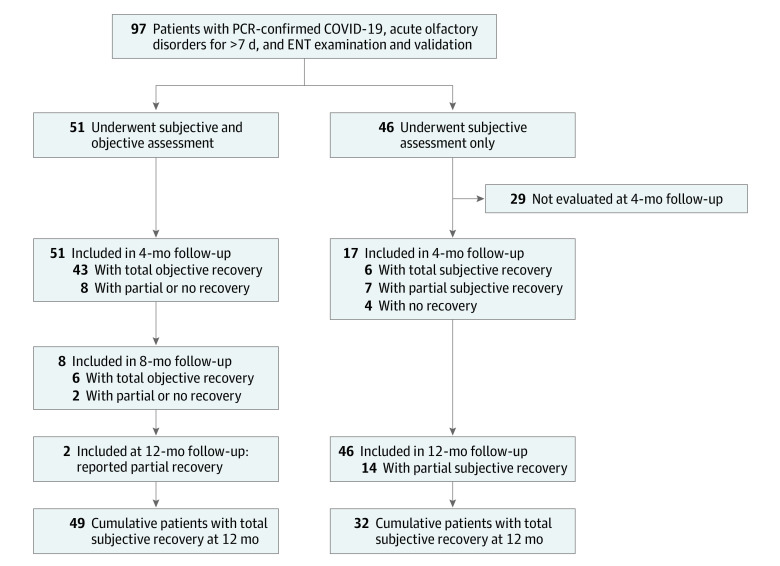
Flowchart of the Study With Major Results From the initial cohort of 97 patients with polymerase chain reaction (PCR)–proven COVID-19 with acute olfactory disorders lasting for more than 7 days, 51 patients were followed up for a year, with subjective and objective olfactory assessment every 4 months, until normalization of objective olfactory test results. ENT indicates ear, nose, and throat.

**Table.** Characteristics of Patients With COVID-19 With Subjective and Objective Olfactory Assessment at 4, 8, and 12 Months

Characteristic	Patients, No. (%)		
	At 4 mo (n = 51)	At 8 mo (n = 8)	At 12 mo (n = 2)
Age, mean (SD), y	38.8 (11.5)	38.6 (16)	48 (18.4)
Sex			
Female	37 (72.5)	8 (100.0)	2 (100.0)
Male	14 (27.5)	0	0
Self-assessment of olfactory loss			
Total	23 (45.1)	6 (75.0)	0
Partial	27 (52.9)	2 (25.0)	2 (100.0)
None	1 (2.0)	0	0
Self-assessment of olfactory recovery time, d		ND	ND
<15	11 (47.8)		
16 to <30	5 (21.7)		
30 to <60	6 (26.1)		
60 to 90	1 (4.4)		
Quantitative olfactory disorder scores ranges			
0-10			
Threshold test	5 (9.8)	1 (12.5)	1 (50.0)
Identification test	5 (9.8)	1 (12.5)	1 (50.0)
11-25			
Threshold test	3 (5.8)	1 (12.5)	0
Identification test	5 (9.8)	1 (12.5)	0
26-50			
Threshold test	9 (17.7)	4 (50.0)	1 (50.0)
Identification test	6 (11.8)	2 (25.0)	0
51-75			
Threshold test	9 (17.7)	1 (12.5)	0
Identification test	7 (13.7)	1 (12.5)	0
76-90			
Threshold test	13 (25.5)	0	0
Identification test	9 (17.7)	2 (25.0)	1 (50.0)
91-95			
Threshold test	5 (9.8)	0/0	0
Identification test	9 (17.7)	0	0
96-100			
Threshold test	7 (13.7)	1 (12.5)	0
Identification test	10 (19.5)	1 (12.5)	0
Qualitative olfactory disorders			
Parosmia (distorted smell)	14 (27.5)	1 (12.5)	1 (12.5)
Fantosmia (olfactory hallucinations)	13 (25.5)	1 (12.5)	0
Persistent COVID-19–related symptoms			
None	34 (66.7)	6 (75.0)	1 (50.0)
Fever	0	0	0
Cough	1 (2.0)	0	0
Respiratory problems	5 (9.8)	0	0
Nasal obstruction	5 (9.8)	0	0
Rhinorrhea	3 (5.9)	1 (12.5)	1 (50.0)
Sinus pain	3 (5.9)	0	0
Headache	7 (13.7)	0	0
Sore throat	0	0	0
Digestive problems	2 (3.9)	0	0
Arthralgia or myalgia	3 (5.9)	0	0
Asthenia	10 (19.6)	1 (12.5)	0
Neurological disorders	0	0	1 (50.0)^a^

Abbreviation: ND, not done.

^a^ Includes memory and planning disorders.

## Discussion

More than 1 year into the pandemic, we describe the long-term prognosis for a cohort of patients with COVID-19–related anosmia, most of whom (96.1%) objectively recovered by 12 months. Our findings suggest that an additional 10% gain in recovery can be expected at 12 months, compared with studies with 6 months of follow-up that found only 85.9% of patients with recovery.^4^ This supports findings from fundamental animal research, involving both imaging studies and postmortem pathology, suggesting that COVID-19–related anosmia is likely due to peripheral inflammation.^4^

We also confirmed that discrepancies exist between self-assessed and objective testing, whereby participants tend to underappreciate the return of normosmia. This highlights the importance of applying both methods for postviral olfactory disorder evaluation.^5^ Discrepancies could be explained by qualitative disorders disrupting self-assessment (eg, parosmia) and/or limited capacity of olfactory tests to capture a complete return to function among individuals with higher baseline olfactory abilities.

The main limitation of our study was that only one-half of the cohort underwent objective olfactory testing. However, all participants were contacted at 12 months and almost all reported a subjective return of smell. It should also be noted that our cohort consisted mainly of women and younger patients (<50 years old), both of which are factors positively associated with full olfactory recovery.^6^

## Conclusions

Persistent COVID-19–related anosmia has an excellent prognosis with nearly complete recovery at 1 year. As clinicians manage an increasing number of people with post-COVID syndrome, data on long-term outcomes are needed for informed prognostication and counseling.
